# Hepatitis C Prevalence Among Iranian Female prisoners

**DOI:** 10.5812/hepatmon.8130

**Published:** 2012-09-30

**Authors:** Fatemeh Abdi

**Affiliations:** 1 Department of Nosocomial Infection Research Center, Isfahan University of Medical Sciences, Isfahan, IR Iran

**Keywords:** Female, Prisoners, Hepatitis C

Dear Editor,

We read with interest the recent report by Nokhodian et al. based on prevalence and risk factors of sexually transmitted infections including hepatitis C infection among female prisoners in Isfahan (1). The researchers successfully presented a picture of the current situation in the prevalence of such important factors in a selected group with specific social behaviors and risk factors. Hepatitis C virus is one of the important causes of hepatitis worldwide. It is also a major cause of cirrhosis and end stage of liver disease (2). Hence reporting the prevalence and its related risk factors in any community is of great importance. Prisoners, as a group with high risk behaviors are at a major risk of being affected with such an infection. Specifically IV drug abusers or those with illegal sex behaviors are among major groups at risk. The article by Nokhodian et al. is successful in presenting a true scenario of what already exists in our prisons in Iran, although their report of the rate of prevalence of HCV infection is very far from other reports from western countries. The prevalence of HCV infection in prisons in Ireland and United States have been reported to be 37% and 34.3%, respectively (3, 4) which is significantly higher than what is reported from Isfahan (7.4%). Both the latter reports emphasized the role of IV drug abuse and high risk sexual behaviors among the prisoners, which is similar to the results by Nokhodian and coworkers; however the reason for the great difference between the HCV prevalence in our prisoners in comparsion with their western counterparts is not determined yet. Although cultural differences that lead to more limited sexual behavior and drug abuse in our community can be a major reason, one other possible reason can be the different sex distribution among the prisoners. In the Iranian article all the prisoners were women (163) in whom IV drug abuse is usually lower than men, while in the Irish report only a small part of the participants (57 out of 1205) were women. This male dominancy may lead to the higher HCV prevalence among those prisoners. Educational programs play a crucial role in creating insight and decreasing the rate of HCV transmission in prisoners. Nokhodian and colleagues recommended that educational programs should be implemented in prison population to decrease the hazardous effects of such infections. Their suggestion regarding the crucial role of training and educational program in patients affected with hepatitis C infection is in agreement with report of La Torre and coworkers who also believe that educational programs can reduce the spread of such an infection (5). In a similar recent study in Isfahan (In press) on patients who had been referred to correctional facilities, we found a similar effect for education compared to Nokhodian' study. Although some of our patients were naturally different from the selected female prisoners with the aforementioned risk factors, the setting of both of our studies was Isfahan with an identical geographical, as well as sociodemographic and cultural pattern. We found that patients with HCV infection will be more informative regarding the nature and risk factors of hepatitis C after effective education. The information level of our patients rose from 11.9 ± 5.3 (before training) to 18 ± 3.13 (after training), which was statistically significant. While only 16.5% of our patients had excellent information about the disease before the educational program, this figure rose to 77.5% after the program which shows a prominent improvement after the educational intervention. Overall the serious hazardous effect of HCV infection warrants appropriate management to reduce its life threatening side effects. Educational measuringes should be the main focus of such a management. By increasing the information level of individuals at risk, more resources are saved and less national budget, which is currently used to treat such patients, can be reserved. 
